# Synthesis of High-Purity Porous Organic Polymers and Exploration of Their Inherent Functionality

**DOI:** 10.1021/acsami.6c00001

**Published:** 2026-03-25

**Authors:** Kohei Okubo, Kotaro Omote, Hitoshi Kasai, Kouki Oka

**Affiliations:** † Institute of Multidisciplinary Research for Advanced Materials, Tohoku University, 2-1-1 Katahira, Aoba-ku, Sendai, Miyagi 980-8577, Japan; ‡ Carbon Recycling Energy Research Center, Ibaraki University, 4-12-1 Nakanarusawa, Hitachi, Ibaraki 316-8511, Japan; § Deuterium Science Research Unit, Center for the Promotion of Interdisciplinary Education and Research, 13101 Kyoto University, Yoshida, Sakyo-ku, Kyoto 606-8501, Japan

**Keywords:** Porous Organic Polymer, Iodine, Chemical Polymerization, Gas Adsorption, Proton Conduction

## Abstract

Porous organic polymers (**POP**s) are porous materials consisting of monomers linked by irreversible covalent bonds, are thermally and chemically stable, and can contain functional backbones at high density. Therefore, **POP**s are suitable for broad applications such as adsorbents and energy storage materials. Typically, **POP**s have been synthesized via chemical polymerization using metal catalysts or oxidants and electropolymerization, although residual impurities and nonuniformity of the material remain significant challenges. This perspective outlines the characteristics of conventional polymerization methods and then introduces a promising method where iodine serves as an oxidant or catalyst (iodine-based chemical polymerization) to synthesize high-purity **POP**s. Finally, based on the inherent functionalities of **POP**s—revealed for the first time through the high-purity synthesis—the future outlook is provided.

## Graphical abstract

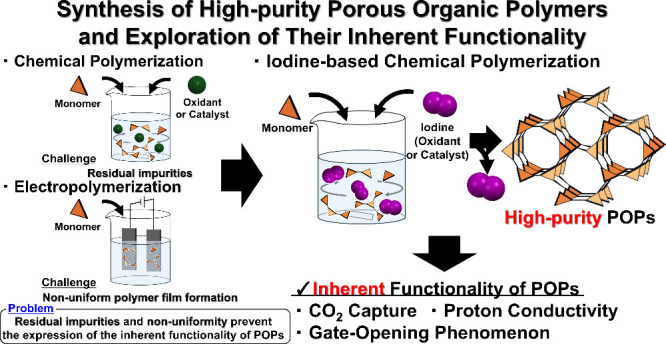

## 1 Introduction

Porous organic polymers (**POP**s)^1^ are composed of only organic molecules linked by strong covalent bonds to form a highly porous framework, and have attracted attention because their constituents are abundant elements and their pore shapes and functions can be tuned by adjusting the molecular design of their monomers.^2,3^ In addition, the metal-free framework of **POP**s allows a lightweight and low-density structure, making it possible to achieve a larger specific surface area (surface area per unit mass) than that of organic–inorganic hybrid porous materials such as metal–organic frameworks^4–8^ (**MOF**s) (Table 1, middle row).^9^ **POP**s are constructed by irreversible covalent bonds between monomers^10^ (Table 1, upper row), and therefore their chemical stability is higher than those of crystalline materials constructed by reversible bonds, such as covalent organic frameworks^11–13^ (**COF**s) and hydrogen-bonded organic frameworks^14–17^ (**HOF**s) (Table 1, bottom row).^10,18^ In addition, **POP**s exhibit high thermal stability, generally retaining their structure at temperatures exceeding 300 °C.^1^ In addition, the synthesis of **POP**s is often achievable via simple mixing procedures, allowing for facile scale-up. Because of their simple structure, **POP**s—apart from the direct covalent connections between their monomeric units—require no additional bonds or functional groups (nodes) for framework formation, which allows the introduction of functional sites at high density. Therefore, **POP**s could be structurally tailored to various applications, such as adsorbents,^19^ energy storage materials,^20^ and sensing materials.^21^

**Table 1:** Comparison of Materials Comprising Porous Frameworks

	Porous Organic Polymers	Metal–Organic Frameworks	Covalent Organic Frameworks	Hydrogen-Bonded Organic Frameworks
Types of bonds	Irreversible covalent bond	Coordination bond	Reversible covalent bond	Hydrogen bond
Mass per unit volume	Low	High	Low	Low
Stability against hydrolysis	High	Low^a^	Low^b^	Low

a Generally sensitive to water.

b Relatively high depending on the linkage (i.e., imine bond).

As shown in Figures 1a and 2, conventionally, **POP**s have been synthesized mainly via chemical polymerization methods using metal catalysts or oxidants (metal-based chemical polymerization), including coupling reactions that require organometallic catalysts^22^ and oxidative polymerization with the aid of oxidants.^23^ These catalysts or oxidants contain metals, which lead to the formation of residual (metal-containing) impurities within the resulting materials. Representative residual metals derived from catalysts or oxidants, such as iron or palladium, often remain within a concentration range of several ppm to several% or lower levels.^24,25^ In contrast, as shown in Figure 1b, electropolymerization has the advantage of yielding high-purity **POP**s, although maintaining a uniform current density on the electrode is difficult. This gives rise to the formation of a nonuniform thin film^26^ and partial overoxidation^27^ of the polymer. Residual impurities that could block the pores in metal-based chemical polymerization^28^ and backbone degradation resulting from overoxidation in electropolymerization^29^ could adversely impact the functionality of the material. Therefore, elucidation of the inherent properties of **POP** materials would require high-purity **POP**s, with metal impurities minimized to negligible levels, e.g., ppb-ppt level, below the detection limit of X-ray photoelectron spectroscopy (XPS) or inductively coupled plasma mass spectrometry (ICP-MS), to be synthesized.

**Figure 1:**
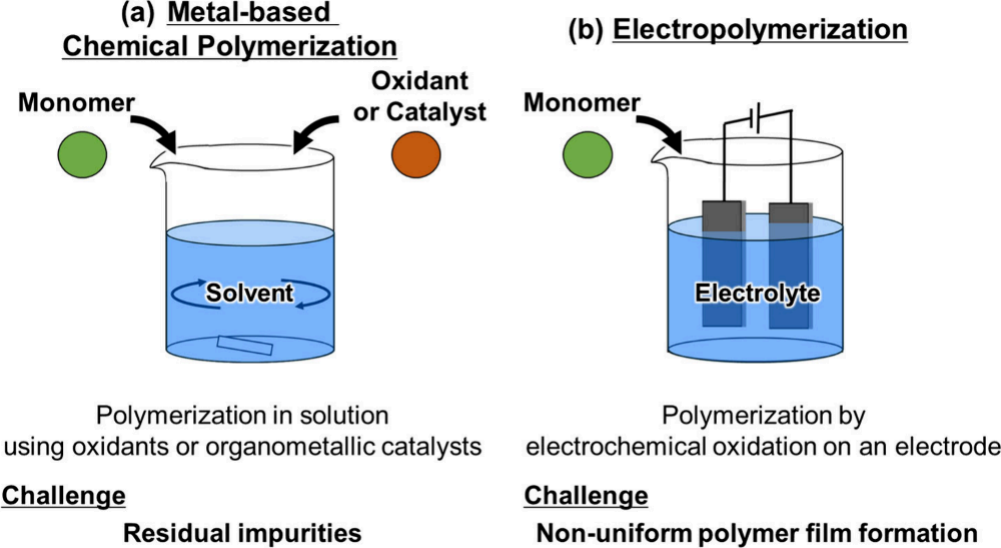
Schematics of (a) metal-based chemical polymerization and (b) electrochemical polymerization.

**Figure 2:**
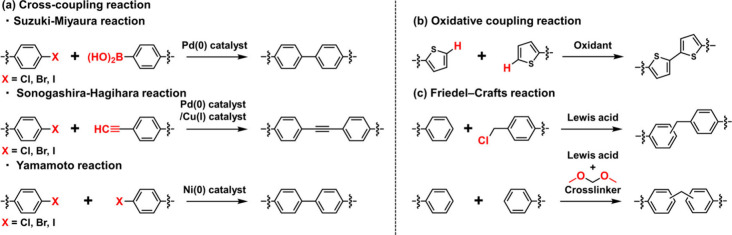
Representative reaction schemes for the synthesis of **POP**s via metal-based chemical polymerization: (a) cross-coupling, (b) oxidative coupling, and (c) Friedel–Crafts reactions.

As shown in Figure 3, this perspective compares and contrasts new and conventional methods to synthesize **POP**s, including porous aromatic frameworks, conjugated microporous polymers, and hyper-cross-linked polymers. First, we review conventional metal-based chemical polymerization and electropolymerization by outlining the characteristics and challenges of each approach. Then, as a novel approach to overcome these challenges and synthesize high-purity **POP**s, we introduce a chemical polymerization method in which iodine is used as an oxidant or catalyst, which has been the topic of ongoing development in recent years.^30–36^ In addition, focusing on the inherent functionalities of the high-purity **POP**s that have been prepared via this method, we highlight the significance of high-purity synthesis. We present examples in which the performance (i.e., the CO_2_ adsorption capacity^35^) was enhanced because of the absence of impurities, and in which inherent functionalities, previously hidden by the presence of impurities, were unveiled (proton conductivity^35^ and the gate-opening phenomenon^36^). Finally, the future outlook for the synthesis and application of high-purity **POP**s is discussed. In contrast to existing reviews that focus on general synthesis methods and applications, this perspective particularly emphasizes high-purity synthesis as a prerequisite for unveiling inherent functionalities that were previously obscured by trace metal impurities.

**Figure 3:**
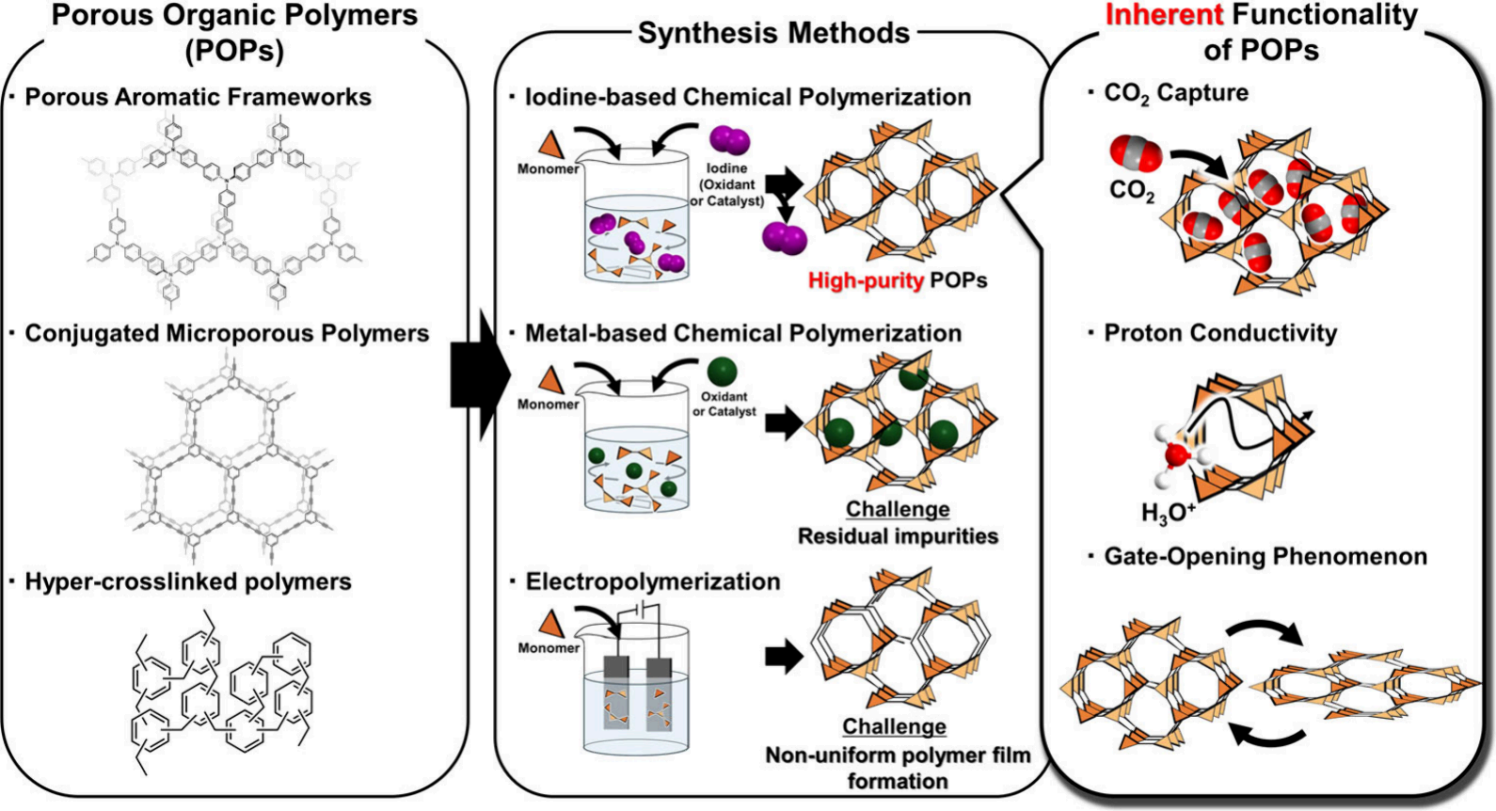
Overview schematic of **POP** classes, synthesis methods, and inherent functionality of **POP**s.

## 2 Polymerization Methods for POPs

As shown in Table 2, the synthesis of **POPs** has conventionally relied on two main approaches: metal-based chemical polymerization^22,37–41^ and electropolymerization.^42^

**Table 2:** Comparison of Polymerization Methods for **POP**s

Method	Metal-Based Chemical Polymerization	Electropolymerization	Iodine-based Chemical Polymerization
Scalability	High	Low	High
Uniformity	High	Low	High
Purity	Low^a^	High	High

a Still contains ppb-level impurities even after many purifications.

### 2.1 Metal-Based Chemical Polymerization

#### Characteristics

Metal-based chemical polymerization is advantageous because it enables the large-scale synthesis of uniform **POP**s by conducting reactions in a solution (even at the kilogram scale^43^) (Table 2, upper row). As shown in Figure 2, representative methods include cross-coupling reactions, oxidative polymerization, and Friedel–Crafts reactions.

#### Cross-Coupling Reaction

As shown in Figure 2a, cross-coupling reactions with organometallic catalysts, represented by the Suzuki–Miyaura coupling,^44^ Sonogashira coupling,^22^ and Yamamoto coupling,^37^ are essential techniques for constructing **POP**s. These reactions enable the construction of diverse structures depending on the combination of monomers.

#### Oxidative Coupling Reaction

Many conjugated **POP**s, such as polytriphenylamine^41^ and polythiophene,^23^ have been synthesized using ferric chloride or aluminum chloride as oxidizing agents. As shown in Figure 2b, these reagents oxidatively couple the aromatic rings of the monomers to form a robust covalent network. The resulting **POP**s are conjugated and possess excellent luminescent and electronic properties.^45^

#### Friedel–Crafts Reaction

As shown in Figure 2c, the Friedel–Crafts reaction relies on Lewis acids such as aluminum chloride as catalysts to react aromatic rings with cross-linkers by linking them via methylene chains or similar structures. This method is particularly widely used for the synthesis of hyper-cross-linked polymers via the self-condensation of aromatic monomers bearing chloromethyl groups^38,40^ or cross-linking reactions in which formaldehyde diacetals serve as external cross-linking agents.^39^

#### Challenges of Metal-Based Chemical Polymerization

Metal-based chemical polymerization is highly scalable, yet a challenge is that residual metal impurities^24,25^ derived from oxidants or catalysts remain in the material (Table 2, bottom row). In particular, small amounts of ferric chloride^24^ (used in oxidative polymerization) and palladium^25^ (the catalyst used in coupling reactions) remain in the resulting **POP**s at ppb-ppm levels. These metal impurities physically block the pores of the **POP**s, which reduces the porosity (the specific surface area and pore volume).^46^ Regarding this point, it is noteworthy that even for frameworks such as porous aromatic frameworks, PAF-1—which exhibit exceptionally high surface areas despite being synthesized via metal catalysts—computational simulations suggest that residual reactants and side-products can cause pore blocking, leading to a loss of experimental porosity compared to theoretical expectations.^47^ In addition, the residual metal impurities themselves potentially act as catalytically active sites^48^ that promote unexpected side reactions and prevent the evaluation of the inherent catalytic functions of **POP**s. However, residual metal impurities sometimes serve as functional components. For example, in the porous polymer COP-220, palladium and copper trapped during synthesis were utilized as active sites for size-selective Suzuki–Miyaura coupling,^49^ demonstrating a sustainable approach in which residual impurities function as embedded catalysts.

### 2.2 Electropolymerization

#### Characteristics of Electropolymerization

A synthesis method that avoids residual metal impurities is electrochemical polymerization.^42^ This method produces **POP**s in the form of thin films by electrochemically oxidizing and polymerizing monomers on an electrode substrate. This method offers the advantage of yielding high-purity **POP**s because it obviates the need for oxidants or catalysts (Table 2, bottom row).

#### Challenges of Electropolymerization

Despite the advantages of electropolymerization (yielding high-purity materials), various challenges would have to be addressed to ensure the method is suitable for practical application. First, maintaining a uniform current density over a large area is intrinsically difficult. Polymerization proceeds preferentially in regions of lower resistance, inevitably leading to nonuniform film formation. The resulting nonuniform current density has the effect of localized overoxidation of **POP**s.^27^ This overoxidation causes degradation, such as decomposition of the backbone of the **POP** (Table 2, middle row), to ultimately degrade the inherent functionality of the material.^29^ Consequently, practical synthesis is restricted to small areas (typically a few cm^2,50^ ) with film thicknesses limited to hundreds of μm to ensure quality, preventing large-scale production (Table 2, top row). Thus, conventional metal-based chemical polymerization leads to residual impurities, whereas electropolymerization has the disadvantages of nonuniformity and problematic scale-up. To develop the inherent functionality of **POP**s, a novel synthesis method has become necessary to overcome these challenges.

### 2.3 Polymerization Methods for High-Purity POPs

Endeavoring to synthesize high-purity **POP**s, we developed a novel chemical polymerization method using iodine as an oxidant.^35,36^ As shown in Figure 4b, this method employs triphenylamine or thiophene derivatives as monomers. Polymerization is accomplished by heating these monomers with iodine in a solvent such as 1,2-dichloroethane. As shown in Figure 4a, iodine serves to oxidize the monomer to generate radical cations, and polymerization proceeds via coupling reactions between these radical cations.^51^ The generation of radicals mediated by iodine is reported to proceed through the formation of a charge-transfer complex between an electron-rich monomer and molecular iodine, followed by a single-electron transfer.^52^ The advantage of this iodine-based chemical polymerization lies in the facile removal of iodine, the oxidant. Previous works demonstrated that polythiophene derivatives synthesized via vapor-assisted polymerization using iodine could be highly purified simply by soaking them with organic solvents such as ethanol, and act as photoelectrode catalysts on their own.^30–34^ Similarly, **POP**s can be washed with ethanol after the reaction to completely remove unreacted iodine and iodine-derived impurities. As shown in Figures 4d and 4e, XPS, ICP-MS, and other spectroscopic measurements confirmed the complete absence of nonmetal species, such as iodine-derived impurities and potential oxidative byproducts.^34^ This result indicates that iodine serves purely as an oxidant and does not remain incorporated within the **POP** framework. This enables the synthesis of high-purity **POP**s without any metal impurities, which was difficult to achieve via conventional metal-based chemical polymerization using metal oxidants. In addition, as shown in Figure 4c, a method involving vapor-assisted polymerization was established. This method, which leverages the sublimation properties of iodine by exposing substrates coated with monomers to iodine vapor, allows the fabrication of high-purity **POP** thin films composed of high-boiling-point monomers, which had previously been difficult to achieve.

**Figure 4:**
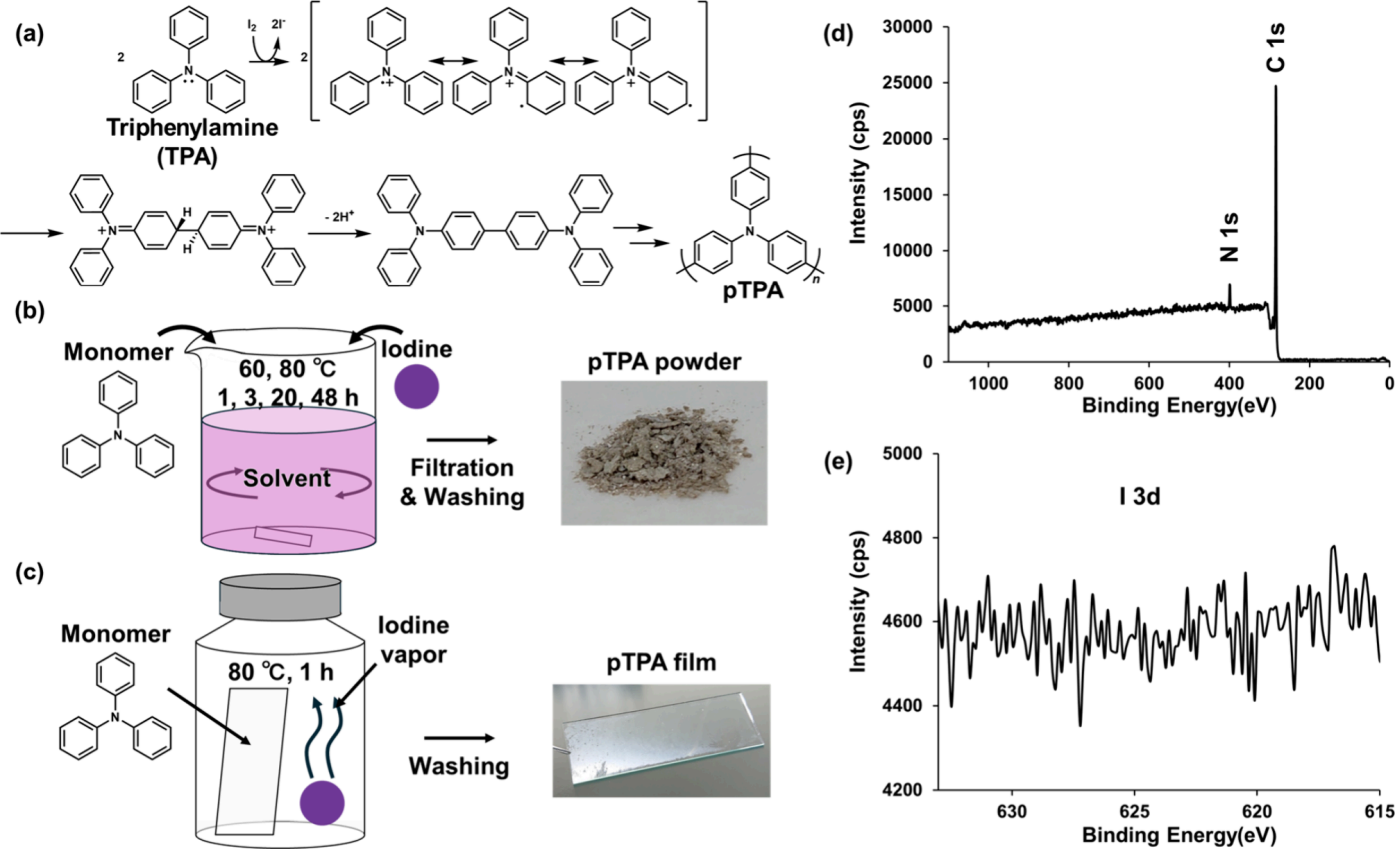
(a) Reaction mechanism of triphenylamine (**TPA**) polymerization using iodine as oxidant and schematics of the procedures for (b) solution polymerization and (c) vapor-assisted polymerization. (d) Full-range XPS spectrum and (e) high-resolution XPS spectrum of the I 3d peaks for polytriphenylamine (**pTPA**). Reprinted with permission from ref 36. Copyright 2025 American Chemical Society.

## 3 Inherent Properties and Potential Applications of High-Purity POPs

The iodine-based chemical polymerization method enabled the synthesis of high-purity **POP**s. By employing triphenylamine-based **POP**s as a representative example, we demonstrate that this high-purity synthesis method makes it possible, for the first time, to explore the inherent properties and potential applications of **POP**s (3.1. CO_2_ Capture, 3.2. Proton Conductivity, 3.3. Gate-Opening Phenomenon), which had previously been masked by metal impurities.

### 3.1 CO_2_ Capture

The gas adsorption capacity of **POP**s is highly dependent on their specific surface area. However, the specific surface area of **POP**s synthesized by conventional metal-based chemical polymerization is difficult to enlarge because the pores are clogged by residual metal impurities. As shown in Figure 5a, our attempt to maximize the porosity of **POP**s involved polymerizing a triphenylamine derivative (**TTPA**). This approach enabled the number of reaction sites to be increased by utilizing a monomer in which three triphenylamine units are prelinked with a benzene core, via iodine-based chemical polymerization without generating residual impurities.^35^ As shown in Figure 5b, the obtained high-purity polytriphenylamine derivative (**pTTPA**) achieved the highest specific surface area (2134.6 m^2^/g) among triphenylamine-based **POP**s (534–1543 m^2^/g^53–55^ ). As summarized in Table 3, this value significantly exceeds that of **pTTPA** synthesized via metal-based chemical polymerization (595 m^2^/g^56^), attributed to the complete elimination of pore clogging caused by metal impurities. As shown in Figure 5c, the outstanding porosity of **pTTPA** increased the CO_2_ adsorption capacity (74.3 mL/g @25 °C, 110 kPa). The increase in CO_2_ uptake is attributed to the maximization of accessible micropores.^57^ In the high-purity **pTTPA**, the value of micropore volume reached 1.51 cm^3^/g. The complete removal of metal impurities, which would otherwise block these subnanoscale pores, allows for enhanced adsorption potentials at low pressures. This result demonstrates that maximization of their specific surface area allows the gas adsorption performance of high-purity **POP**s to be increased drastically. In addition, the nitrogen-rich triphenylamine units enhance CO_2_ affinity through dipole–quadrupole interactions, the high-purity synthesis plays a complementary and crucial role by ensuring these functional sites are not obscured by residual metal species. This synergy between chemical functionality and structural purity maximizes the intrinsic adsorption potential of the material. The long-term stability and reproducibility of the CO_2_ capture performance are ensured by the rigid covalent-bonded structure of the **POP** frameworks and the intrinsic reversibility of the physisorption mechanism. This structural robustness is evidenced by the reproducibility of N_2_ adsorption isotherms at 77 K, which remained identical even after repetitive high-temperature vacuum degassing. Since these characterization conditions are more rigorous than those required for CO_2_ desorption, the high-purity **POP**s are expected to maintain stable performance and high durability over extended cycling.

**Figure 5:**
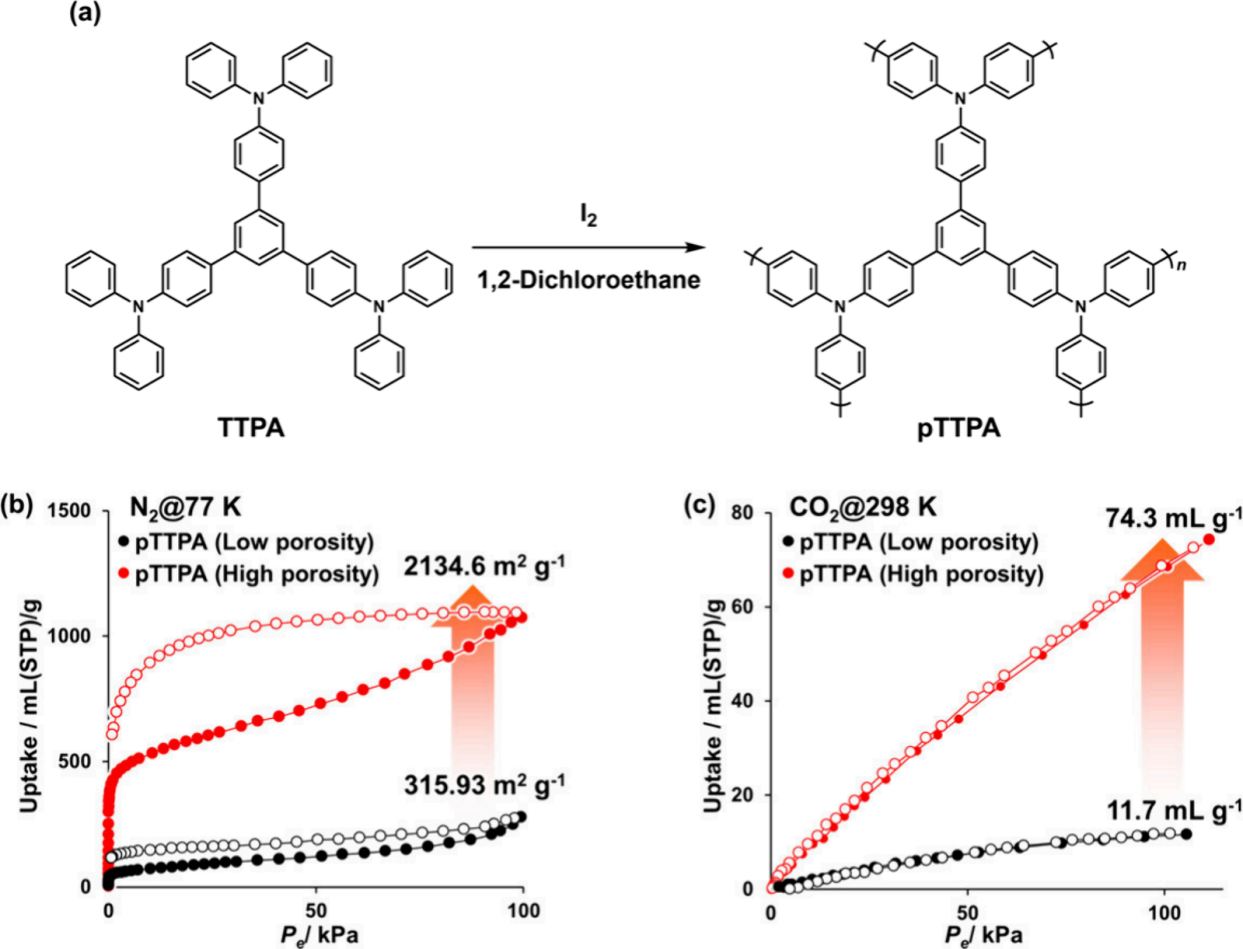
Schematics of (a) **pTTPA** polymerization and gas adsorption isotherms of (b) N_2_ (77 K) and (c) CO_2_ (298 K) of **pTTPA**s. The solid and open symbols denote the adsorption and desorption isotherms, respectively. Reproduced under terms of the CC-BY license.^35^ Copyright 2025, K. Okubo, S. Kitajima, H. Kasai, K. Oka, published by Wiley-VCH.

**Table 3:** Comparison of Polytriphenylamine Derivatives Synthesized Using Iodine or Metals as an Oxidant

Sample	Oxidant	Specific Surface Area (m^2^/g)	Properties	Reference
High-purity **pTPA**	Iodine	(2.7 ± 0.2) × 10^2^ ^a^	Gate-opening behavior	^36^
Conventional **pTPA**	FeCl_3_	1437		^54^
High-purity **pTTPA**	Iodine	2134.6	High CO_2_ adsorption capacity/High proton conductivity	^35^
Conventional **pTTPA**	FeCl_3_	595		^56^

a Note that the specific surface area of high-purity **pTPA** is underestimated because it was calculated based on adsorption isotherms of the closed-pore structure prior to gate opening.

### 3.2 Proton Conductivity

The maximized porosity that was realized by synthesizing high-purity **POP**s also led to the expression of novel functionalities. Conventionally, the polytriphenylamine backbone itself was considered to be hydrophobic and insulating without any ionic conductivity. However, as shown in Figure 6a, the high-purity polytriphenylamine derivatives synthesized via iodine-based chemical polymerization were found to absorb large amounts of water vapor (up to 32.8 mmol/g) under high humidity (95% RH) conditions as a result of their expansive specific surface area.^35^ Consequently, as shown in Figure 6b, the polytriphenylamine derivative, which had previously been considered to be an insulator, was measured to have proton conductivity of 4.33 × 10^–6^ S cm^–1^ (@ 95% RH and 90 °C). This conductivity originates from the synergy of heteroatom exposure and pore size distribution. High surface area increases the density of nitrogen atoms on the pore walls, which serve as nucleation sites for water clusters.^58^ In addition, high microporosity enhances the confinement effect,^59^ lowering the activation energy for water self-dissociation and promoting proton transport preferentially mediated by the vehicle mechanism. The fact that this conductivity is 3 orders of magnitude higher than that of low-porosity **POP**s indicates that maximizing the porosity by synthesizing high-purity **POP**s led to the expression of potential functionality.

**Figure 6:**
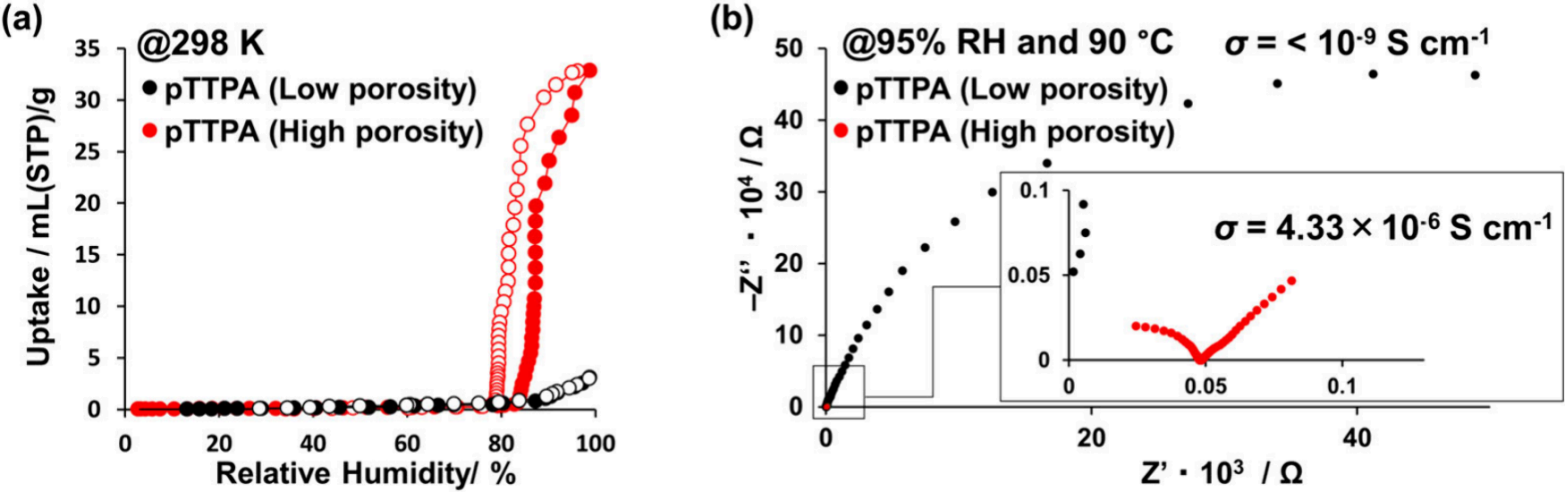
(a) Water–vapor adsorption isotherms of **pTTPA**s, measured at 298 K. Solid and hollow symbols denote the adsorption and desorption isotherms, respectively. (b) Proton conductivities of **pTTPA**s. Impedance spectra of the disk-shaped pellets of the two types of **pTTPA** at 95% RH and 90 °C. Reproduced under terms of the CC-BY license.^35^ Copyright 2025, K. Okubo, S. Kitajima, H. Kasai, K. Oka, published by Wiley-VCH.

### 3.3 Gate-opening Phenomenon

Another property that emerged as a result of high-purity synthesis via iodine-based chemical polymerization is the inherent flexibility of **POP**s. Conventionally, **POP**s, which are constructed by forming irreversible covalent bonds, were considered to possess a rigid network structure. However, as shown in Figure 7, the high-purity polytriphenylamine synthesized via iodine-based chemical polymerization was discovered to exhibit the gate-opening phenomenon^60,61^ in N_2_ adsorption measurements, despite its structure consisting of directly bonded rigid triphenylamine units.^36^ Because of this phenomenon, the amount of N_2_ adsorbed at a specific measurement pressure (90 kPa) increases drastically. This unique behavior, which has thus far been observed only in certain flexible crystalline materials such as **MOF**s^62,63^ and **COF**s,^64,65^ is rarely triggered by gases that engage in weak interactions with adsorbents, such as nitrogen. This is the first observation of the nitrogen-induced gate-opening phenomenon in **POP**s. This result suggests that **POP**s are sufficiently flexible to dynamically transform their pore structure in response to stimuli. As shown in Figure 7b, *in situ* IR measurements under nitrogen atmosphere revealed intensity changes in the 1500–1600 cm^–1^ region at 98 kPa, suggesting a dynamic expansion of the framework as it transitions from a closed- to an open-pore state. In contrast, **POP**s that are synthesized by conventional metal-based chemical polymerization are prevented from exhibiting dynamic behavior by the residual metal impurities, suggesting that this inherent property of the material was previously overlooked.

**Figure 7:**
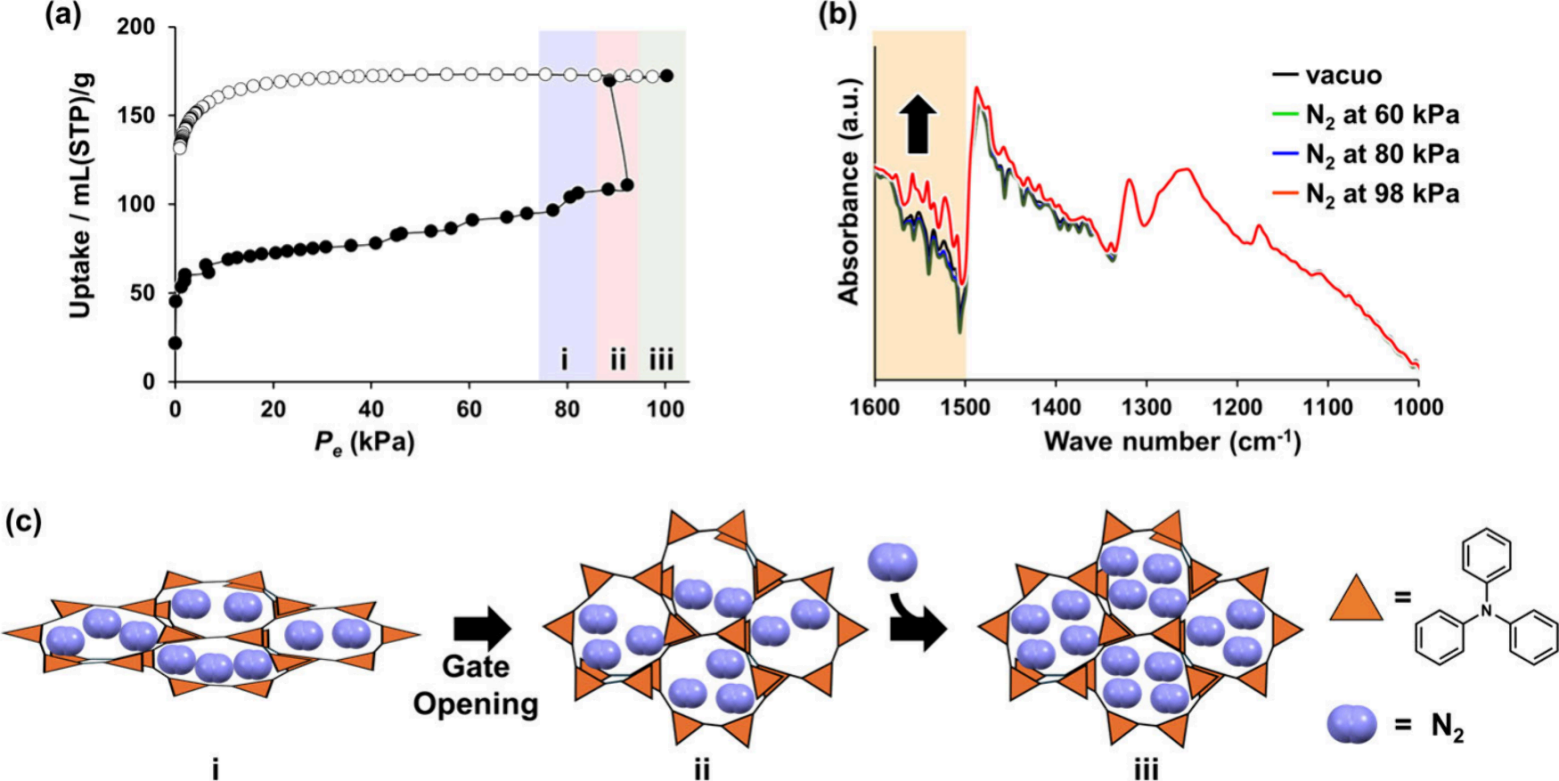
(a) N_2_ adsorption isotherms of **pTPA**, measured at 77 K. Solid and open symbols denote adsorption and desorption isotherms, respectively. (b) *In situ* IR spectra recorded for **pTPA** under vacuum (black), 60 kPa (blue), 80 kPa (green), and 98 kPa (red) N_2_ atmospheres at 77 K. The orange area represents the range of wavenumbers with increased peak intensity. (c) Schematics of the structural transition of **pTPA** induced by N_2_ adsorption. Reprinted with permission from ref 36. Copyright 2025 American Chemical Society.

As summarized in Table 4, high purity is essential for maximizing the functional potential of **POP**s. Beyond gas adsorption and proton conduction, the detrimental impact of residual impurities extends far beyond structural characterization, interfering with a wide range of physical and functional properties, including thermal stability,^66^ framework flexibility,^36^ and optical performance.^67^ These examples highlight that achieving high purity is a critical requirement for exploring the intrinsic properties of **POP**s.

**Table 4:** Impact of Residual Impurities on Characterization and Performance

Aspect	Interference Mechanism	Impact on Observation
Thermal stability	Catalytic promotion of decomposition	Underestimation of intrinsic thermal stability due to accelerated degradation.
Porosity	Physical pore-blocking	Underestimation of specific surface area and pore volume
Flexibility	Structural anchoring	Suppression of dynamic framework movement and gate-opening behavior
Proton transport	Disruption of conduction paths	Lowered proton conductivity due to hindered water mobility in blocked pores
Optical property	Fluorescence quenching	Reduced quantum yield and shortened excited-state lifetime hinder applications in sensing, photocatalysis, and optoelectronics
Catalytic activity	Metal-derived catalytic activity or active site poisoning	Misleading evaluation: overestimation derived from catalytic activity of trace impurities or underestimation of inherent activity through poisoning

## 4 Conclusion and Future Outlook

This perspective outlines synthesis methods for **POP**s with a particular focus on the development of high-purity materials. The challenges associated with conventional metal-based chemical polymerization and electropolymerization methods (residual metal impurities, nonuniformity, and scale-up issues) can be overcome by using iodine-based chemical polymerization, a novel approach that entails the use of iodine as an oxidant. This method enables the uniform, large-scale synthesis of high-purity **POP**s without any metal impurities, in both powder and thin film forms. These high-purity **POP**s have various advantageous inherent properties, among which (1) increased CO_2_ adsorption capacity as a result of maximized porosity, (2) proton conductivity despite being insulating materials, and (3) gate-opening phenomena during N_2_ adsorption. Iodine-based chemical polymerization is expected to be applicable not only to triphenylamine-based monomers but also to diverse electron-rich aromatic monomers that were conventionally synthesized via oxidative polymerization, such as thiophene, pyrrole, and carbazole derivatives. This high replicability suggests that the method can be generalized to a wide range of **POP** paradigms. However, a potential limitation of this method is its dependence on the oxidation potential of iodine (0.54 V vs SHE^34^). Since the polymerization initiates with the one-electron oxidation of monomers to generate radical cations, its applicability is currently focused on electron-rich aromatic building blocks. Consequently, electron-deficient monomers that possess high oxidation potential remain challenging to polymerize using iodine as an oxidant. To address this limitation, future research strategies should focus on the selection of nonmetal oxidants appropriate for the oxidation potential of monomers. For monomers with high oxidation potentials, the use of stronger nonmetal oxidants with higher oxidation potentials, such as nitrosonium hexafluorophosphate (1.51 V vs SHE^68^), may provide an alternative metal-free route. Tailoring the nonmetal-based system to the monomer’s electronic properties will be a key strategy for expanding the library of high-purity **POP**s. In addition, as shown in Figure 8, the role of iodine extends beyond the function of an oxidant, as discussed in this perspective (Figure 8, blue). For example, the ability of iodine to act as a Lewis acid suggests potential applications as a replacement for metal catalysts (such as aluminum chloride) in conventional Friedel–Crafts reactions^69^ (Figure 8, green) and as a catalyst in other polymerization reactions such as Glaser coupling^70^ (Figure 8, red). Integrating the versatile reactivity of iodine with the strategic selection of other nonmetal systems will establish a versatile and robust platform for constructing a wide variety of high-purity **POP**s.

**Figure 8:**
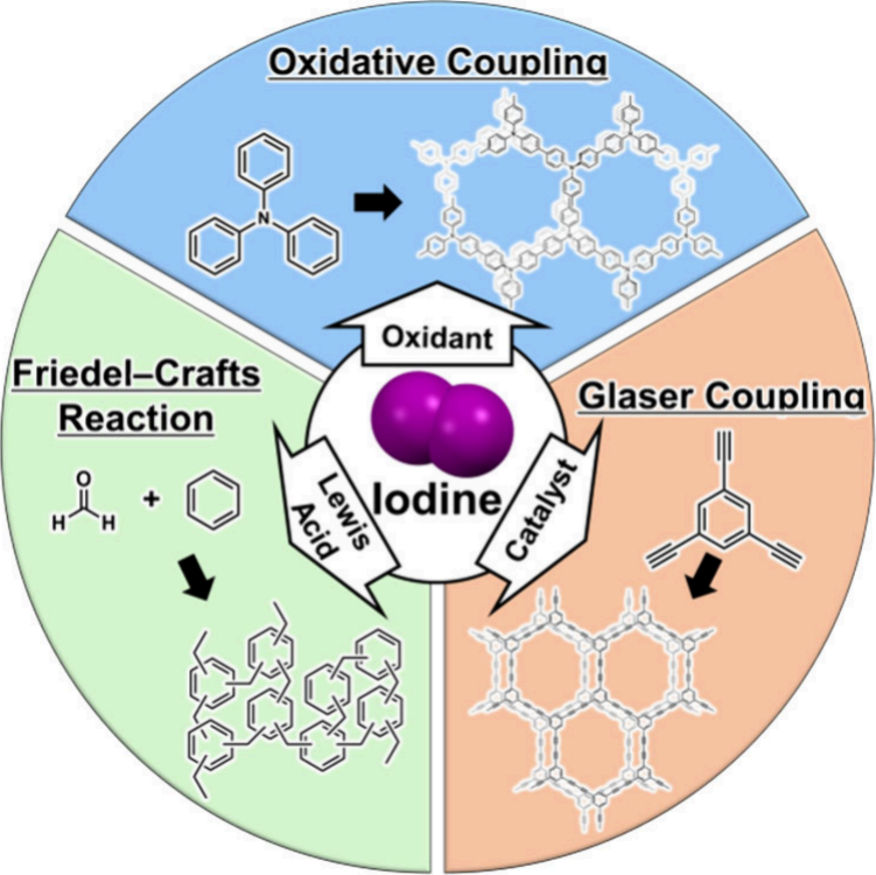
Outlook for the synthesis of high-purity **POP**s based on the diversified role of iodine.

This novel chemical polymerization method, which exploits the diverse reactivity of iodine, further broadens the synthesis possibilities of high-purity **POP**s without residual impurities. In ongoing work, this approach could be extended to other **POP**s and polymer materials to enable us to re-evaluate and explore the inherent functionalities within the existing material groups through high-purity purification. In other words, polymers still hold unprecedented potential for developing novel functionalities.

## ABBREVIATIONS

POPs: porous organic polymers
MOFs: metal–organic frameworks
COFs: covalent organic frameworks
HOFs: hydrogen-bonded organic frameworks
pTPA: polytriphenylamine
